# The Repetitive Domain of ScARP3d Triggers Entry of *Spiroplasma citri* into Cultured Cells of the Vector *Circulifer haematoceps*

**DOI:** 10.1371/journal.pone.0048606

**Published:** 2012-10-31

**Authors:** Laure Béven, Sybille Duret, Brigitte Batailler, Marie-Pierre Dubrana, Colette Saillard, Joël Renaudin, Nathalie Arricau-Bouvery

**Affiliations:** 1 INRA, UMR 1332 Biologie du Fruit et Pathologie, Villenave d'Ornon, France; 2 Université de Bordeaux, UMR 1332 Biologie du Fruit et Pathologie, Villenave d'Ornon, France; 3 Université de Bordeaux, UMS3420, Bordeaux Imaging Center, Bordeaux, France; 4 CNRS, Bordeaux Imaging Center, UMS 3420, Bordeaux, France; 5 INSERM, Bordeaux Imaging Center, US 004, Bordeaux, France; Institut Pasteur Paris, France

## Abstract

*Spiroplasma citri* is a plant pathogenic mollicute transmitted by the leafhopper vector *Circulifer haematoceps*. Successful transmission requires the spiroplasmas to cross the intestinal epithelium and salivary gland barriers through endocytosis mediated by receptor-ligand interactions. To characterize these interactions we studied the adhesion and invasion capabilities of a *S. citri* mutant using the Ciha-1 leafhopper cell line. *S. citri* GII3 wild-type contains 7 plasmids, 5 of which (pSci1 to 5) encode 8 related adhesins (ScARPs). As compared to the wild-type strain GII3, the *S. citri* mutant G/6 lacking pSci1 to 5 was affected in its ability to adhere and enter into the Ciha-1 cells. Proteolysis analyses, Triton X-114 partitioning and agglutination assays showed that the N-terminal part of ScARP3d, consisting of repeated sequences, was exposed to the spiroplasma surface whereas the C-terminal part was anchored into the membrane. Latex beads cytadherence assays showed the ScARP3d repeat domain (Rep3d) to be involved, and internalization of the Rep3d-coated beads to be actin-dependent. These data suggested that ScARP3d, via its Rep3d domain, was implicated in adhesion of *S. citri* GII3 to insect cells. Inhibition tests using anti-Rep3d antibodies and competitive assays with recombinant Rep3d both resulted in a decrease of insect cells invasion by the spiroplasmas. Unexpectedly, treatment of Ciha-1 cells with the actin polymerisation inhibitor cytochalasin D increased adhesion and consequently entry of *S. citri* GII3. For the ScARPs-less mutant G/6, only adhesion was enhanced though to a lesser extent following cytochalasin D treatment. All together these results strongly suggest a role of ScARPs, and particularly ScARP3d, in adhesion and invasion of the leafhopper cells by *S. citri*.

## Introduction

*Spiroplasma citri* is a phloem-limited plant pathogenic bacterium belonging to the class *Mollicutes* and was the first plant mollicute to be cultivated in cell-free medium [1]. *S. citri* is typically known as the causal agent of citrus stubborn and horseradish brittle root diseases [2], [3] but it also infects many other plants including cruciferous, carrot, and periwinkle. In nature, transmission of *S. citri* from infected to healthy plants is mediated by phloem sap-feeding leafhoppers, *Circulifer haematoceps* in the Mediterranean basin [4] and *Circulifer tenellus* in the USA and Mediterranean basin [5], [6], in a persistent propagative manner. Successful transmission requires the spiroplasmas to pass through enterocytes of the mid-gut epithelium, multiply to high population in the hemocoel, and invade the salivary glands to be released in the saliva duct [7], [8]. Transmission electron microscopy (TEM) studies investigating invasion of insect cells *in vivo* and *ex vivo* both revealed invagination of the cell membrane in close contact with the spiroplasmas [9], [10], [11], [12], [13]. Together with the presence of spiroplasmas within membrane-bound cytoplasmic vesicles of insect cells [11], these observations strongly suggest an endocytosis mechanism mediated by receptor-ligand interactions [8]. During the invasion process, spiroplasma surface proteins are expected to play a key role in the early stage of adherence. In *S. citri*, proteome comparison between the wild-type, insect-transmissible strain GII3 and the non-insect-transmissible strain 44 led to the identification of proteins tentatively associated with insect-transmissibility [13]. In *S. citri* GII3, these proteins are encoded by plasmids pSci1 to 6 [14], which are absent in *S. citri* 44 [15]. The implication of plasmid-encoded determinants in insect-transmission was further documented by the finding that pSci6 from *S. citri* GII3 partially restored insect-transmissibility when introduced into *S. citri* 44 [16]. Similarly, the *S. citri* mutant G/6, which lacks plasmids pSci1 to 5, was less efficiently transmitted than the wild-type strain GII3 [17]. In summary, at the same time as pSci6 contains sequences that are essential for transmission, pSci1 to 5 encode determinants that are required for efficient transmission of *S. citri* by its leafhopper vector *C. haematoceps* [16], [17].

In silico analyses of *S. citri* GII3 plasmids revealed pSci1 to 5 to encode eight *S. citri* adhesion-related proteins (ScARPs) sharing high sequence homology, with overall amino acid identities ranging from 35 to 83%, and conserved domains [14]. In particular, they all possess an N-terminal signal peptide and a putative transmembrane segment at the C-terminal end. With the exception of ScARP4a, the ScARPs also possess a sarpin domain comprising 6 to 8 repeated sequences of 39 to 42 amino acids. Such a domain was first described in the *S. citri* BR3 protein SARP1, which has been tentatively associated with adhesion of spiroplasmas to insect cells in culture [18], [19]. In these studies, limited proteolysis of spiroplasmas resulted in a decreased amount of SARP1 and a concomitant decrease of cytadherence. Conversely, renewal of detectable amounts of SARP1 restored adherence to insect cells. The putative implication of ScARPs in invasion of the eukaryotic insect cells was further reinforced by the fact that they share significant similarity with the *Mycoplasma agalactiae* adhesin P40 known to play a crucial role in cytadherence to lamb synovial cells [20]. However evidence of a direct interaction of ScARPs with the insect cell membrane has not been established. Previously, we showed that *S. citri* 44, a non-insect-transmissible strain lacking ScARPs, was impaired in its ability to invade the leafhopper cell line Ciha-1 [12]. This apparent correlation between the ability of the spiroplasma to invade insect cells *ex vivo* and its ability to be transmitted by the leafhopper vector prompted us to further use the Ciha-1 cell line to explore the implication of ScARPs in the internalization process, with the aim to better understand the function of these adhesins in insect transmission of *S. citri*.

Given that ScARP3d contains all identified domains found in the different ScARPs and possesses one of the largest sarpin domain (7 repeated sequences), it was chosen to investigate the role of ScARPs, in adhesion and entry of *S. citri* into Ciha-1 cells. Using a proteolysis approach, we first confirmed ScARP3d to be surface exposed. We also showed the ability of the ScARP3d repeat domain (Rep3d) to trigger internalization of latex beads into the insect cells. The implication of ScARP3d in the internalization of *S. citri* into the leafhopper cells was further confirmed through inhibition and competitive assays using anti-ScARP3d antibodies and recombinant proteins, respectively.

## Materials and Methods

### Bacterial Strains and Insect Cell Line

*Spiroplasma citri* strain GII3 was originally isolated from its leafhopper vector *C. haematoceps* captured in Morocco [21]. The low-passage, wild-type strain can be experimentally transmitted to plants through feeding/injection to its leafhopper vector and contains seven plasmids pSciA and pSci1 to pSci6 [14]. The *S. citri* GII3 mutant G/6 was engineered through plasmid incompatibility curing. It lacks plasmids pSci1 to 5 encoding the ScARPs [17]. Spiroplasmas were cultivated at 32°C in SP4 medium [22] from which the fresh yeast extract was omitted. *Escherichia coli* strains DH10B and BL21 (DE3) were grown in Luria-Bertani (LB) medium and used to clone and express the *S. citri* recombinant protein His_6_-Rep3d, a truncated form of ScARP3d.

The non-phagocytic cell line Ciha-1 established from *C. haematoceps* [12] was cultured at 32°C as described previously [12].

### Expression and Purification of Recombinant Protein His_6_-Rep3d, and Cleavage of the His_6_-tag

The DNA fragment (912 bp long) corresponding to the ScARP3d repeat region (aa 24–328) was PCR amplified from the *S. citri* GII3 plasmid pSci2 [14] with primers Rep3dXbaF (5′-ATG CAT **TCT AGA** AAC ACA CCC GCA ACA CGC ACC-3′) and Rep3dBamR (5′-TAC TA**G GAT CC**T TAA ACT AAG TTA AAC TTA CTT TGT G-3′); the introduced restriction sites are in bold. The expected 912-bp DNA fragment was digested by *Xba*I and *Bam*HI and ligated to the pET28a(+) vector (Novagen) digested by *Nhe*I+*Bam*HI. The mixture was used to transform *E. coli* DH10B and the transformants were selected on LB medium containing kanamycin (50 µg/ml). The recombinant plasmid pET-Rep3d containing the Rep3d insert was verified by sequencing.

Expression and purification of *S. citri* Rep3d tagged with hexa histidine (His6) at its N-terminus were carried out under native conditions as previously described [23]. The purified His_6_-Rep3d was desalted using a PD-10 column (GE Healthcare) and re-purified on His-select Nickel affinity gel packed column (Sigma). Protein concentration was estimated by the Bradford procedure. Purification was confirmed by 12.5% SDS-PAGE (Fig. S1A). The His_6-_tag was removed using the Thrombin Clean Cleave™ kit (Sigma-Aldrich) according to the manufacturer’s instructions and the resulting recombinant protein Rep3d was verified as above by 12.5% SDS-PAGE (Fig. S1B) and western immunoblotting using anti-Rep3d PAbs as primary antibodies (Fig. S1C) as described below.

### Production of Polyclonal Antibodies and Western Immunoblotting

Rabbit polyclonal antibodies (PAbs) raised against recombinant Rep3d were produced by Covalab SAS (Villeurbanne, France). Specificity to ScARPs of anti-Rep3d PAbs was confirmed by western immunoblotting of crude protein extracts from *S. citri* GII3 and the ScARP-less mutant G/6 (Fig. S1C). Rabbit anti-P32 PAbs were generated by Eurogentec SASU (Angers, France) against individual P32 protein spots obtained by 2D-PAGE as described previously [13]. Rabbit anti-spiralin PAbs were a kind gift from Pr. H. Wróblewski, University of Rennes I, France. Rabbit anti-fibril PAbs were kindly provided by Dr. D.L. Williamson, State University of New-York, USA. To perform western immunoblotting experiments using PAbs as primary antibodies, proteins were separated by 12.5% or 10% SDS-PAGE [24] before being transferred onto a nitrocellulose membrane using a semi-dry apparatus. Proteins were probed using anti-Rep3d, anti-spiralin, or anti-P32 PAbs as the primary antibodies, and goat anti-rabbit immunoglobulin G-peroxidase conjugates (1∶20000 dilution, Sigma) as the secondary antibodies. Immunoreactive proteins were visualized by using the Super Signal West Pico chemiluminescent substrate according to the manufacturer (Pierce).

### Spiroplasma Agglutination Assays Using Polyclonal Antibodies

Exponentially growing GII3 and G/6 spiroplasma strains were collected by centrifugation (12000 g, 4°C, 30 min) and washed with ice-cold Hepes sucrose buffer containing 8 mM Hepes and 280 mM sucrose (HS), pH 7.4. The pelleted spiroplasmas were re-suspended in HS buffer to reach a titer of 10^11^ colony forming units (CFU)/ml. Diluted (1∶10) pre-immune serum, purified anti-spiralin or anti-Rep3d PAbs at concentrations ranging from 0.01 mg/ml to 0.5 mg/ml were added to the cell suspension and incubated at room temperature for 30 min. Anti-spiralin PAbs recognizing the major spiroplasma surface protein [25] were used as the positive control. After removal of excess antibody by centrifugation (12000 g, 4°C, 30 min), the pelleted spiroplasmas were then dispersed in the initial volume of HS buffer and the number of free spiroplasmas (not included in aggregates) was determined by darkfield light microscopy observation. For each sample, free spiroplasmas from10 distinct fields were counted and the resulting number compared to the control. The agglutinating activity was expressed as the minimum immunoglobulin concentration agglutinating 50% of the spiroplasmas (AC_50_).

### Limited Proteolysis Assays

Proteolysis protection assays were performed to assess the surface-exposure of the ScARPs repeated domains. Exponentially growing spiroplasmas were collected by centrifugation (12000 g, 4°C, 30 min) and washed with ice-cold HS buffer. For trypsin proteolysis assays, 10^10^ spiroplasmas were re-suspended in 100 µl of 50 mM sodium phosphate buffer containing 550 mM sorbitol, pH 8.3. The proteolysis was performed using immobilized trypsin (Sigma) at 1 mg/ml at 37°C for 1 h, 4 h, and 24 h under gentle shaking. For proteinase K digestion assays, 10^10^ spiroplasmas were incubated for 10 min at 37°C in 100 µl HS buffer containing immobilized proteinase K (Promega) at different concentrations ranging from 8 to 75 U/ml. At the end of the incubation period, proteolytic enzymes were removed by centrifugation (3000 g, 20°C, 3 min). The proteins recovered in the supernatant were solubilized in SDS-PAGE loading buffer, and boiled at 100°C for 2 min before being separated by 10% SDS-PAGE and detected by western immunoblotting. Proteins were labeled using the monoclonal antibody (MAb) 10G3 [15] followed by goat anti-mouse immunoglobulin G-peroxidase conjugated secondary antibodies (1∶20000 dilution, Sigma), or using anti-Rep3d PAbs or anti-P32 PAbs as described above. The hydrophilic P32 protein, formerly characterized as a major cytoplasmic protein [13], served as the cytoplasmic, protease-inaccessible control.

### Adhesion and Invasion Assays

Adhesion and invasion assays were carried out essentially as described previously [12]. Briefly, *S. citri* (about 5×10^6^ CFU) were added to 10^5^ Ciha-1 cells [multiplicity of infection (MOI) ranging from 26 to 65 depending on the experiments] in a well of 24-well plates with 0.5 ml of insect cell culture medium. After 4 h of contact and 3 washes, cells with adherent spiroplasmas were removed from the well by trypsinization and adequate dilutions were plated on SP4 agar medium. In this case, CFU counts corresponded to the number of cells with associated spiroplasmas, *i.e.* cells with adherent and/or internalized spiroplasmas. In the gentamicin protection assays used to determine the number of infected cells, the gentamicin treatment (400 µg/ml for 3 h) was applied 4 h after adding the spiroplasmas to the Ciha-1 cells. After 3 washes, the leafhopper cells were trypsinized and plated for CFU determination. In this case, CFU counts corresponded to cells with internalized spiroplasmas, which survived the gentamycin treatment. The number of cells with adherent spiroplasmas was calculated by subtracting CFU counts of infected cells from CFU counts of cells with associated spiroplasmas.

For antibody inhibition assays, spiroplasmas were pre-incubated with anti-Rep3d PAbs or anti-fibril PAbs at 32°C for 1 h before they were added to the Ciha-1 cells. Adhesion and invasion were determined as described above.

For competitive assays, Ciha-1 cells were pre-incubated at 32°C for 1 h in the presence of various concentrations (0.4 to 1.5 nmol/well) of recombinant Rep3d or BSA (fraction V Sigma, used as the control). After 3 washes cells were then inoculated with spiroplasmas.

To determine whether cell invasion was an actin-dependent process, the effect of cytochalasin D that inhibits actin polymerization was assessed. Cells were pre-incubated with the drug (0.1 to 10 µg/ml) for 1 h prior to inoculation with spiroplasmas. In this case, cytochalasin D was maintained in the medium during inoculation.

### Coated Latex Beads Binding and Internalization Assays

The yellow-green fluorescent and amine-modified latex beads (4×10^9^ beads of 1 µm) (Sigma) were covalently coated with 64 µg of recombinant Rep3d or BSA according to the supplier's instructions. About 10^5^ cells cultivated on cover slips in 24-well plates were incubated with 2.10^6^ coated latex beads in Schneider medium for 1 h at 32°C. All further steps were carried out at room temperature. After 3 washes in Schneider medium, the cells were fixed for 15 min with 4% paraformaldehyde in PBS and the Rep3d-coated beads were incubated with anti-Rep3d PAbs diluted 1∶500 in PBS-BSA solution (PBS containing 1% BSA) for 30 min. After 3 washes with PBS, the cells were incubated for 30 min with Alexa 633-conjugated goat anti-rabbit antibodies (Invitrogen) diluted 1∶200 and Alexa 568-phalloidin (Invitrogen), used to stain the actin filaments of the Ciha-1 cells, diluted 1∶200 in PBS-BSA buffer. As a result, only extracellular Rep3d-coated beads were stained with Alexa 633.

To visualize internalized beads, a second immunofluorescent staining was performed in parallel in a distinct well. After incubation with coated beads and fixation, the cells were permeabilized by treatment with 0.2% Triton X-100 in PBS-BSA for 15 min, and stained as described above. In this case, both intra- and extracellular Rep3d-coated beads were stained. In control experiments the anti-Rep3d antibodies were omitted to confirm the staining specificity. The cover slips were mounted in the anti-fading ProLong Gold Reagent (Invitrogen) and immunofluorescent samples were imaged using a TCS SP2 upright Leica confocal laser scanning microscope (CLSM), with a 63×oil immersion objective lens with pixel size of 70 nm. Fluorochromes were detected sequentially frame by frame. The images were coded cyan (Alexa 633), green (fluorescent latex beads), and red (Alexa 568). For each experiment 20 to 25 fields with approximately 100 cells per field were observed randomly. The area of single beads was calculated field by field from confocal stack projections for each acquisition with the free software package ImageJ (http://imagej.nih.gov/ij/). The average area of the beads was used to calculate the number of beads per field that was then divided by the number of cells per field. The percentage of internalized latex beads was calculated field by field from confocal stack projections by subtracting the numbers of beads coloured in cyan from those coloured in green, in the experiments without cell permeabilization.

The percentage of internalized coated beads was also determined in the presence of cytochalasin D from confocal stack images. The cells were first pre-incubated with cytochalasin D for 1 h and then addition of the Rep3d-coated latex beads was performed as described above. For the adhesion inhibition assays, the coated latex beads were pre-incubated with anti-Rep3d and anti-spiralin (control) PAbs at room temperature for 30 min, collected by centrifugation at 5 000 rpm for 3 min, and re-suspended in Schneider medium prior to being added to the Ciha-1 cells.

### Transmission Electron Microscopy (TEM)

For transmission electron microscopy, about 10^5^ cells cultivated on cover slips in 24-well plates were incubated with 2.10^6^ Rep3d-coated latex beads in Schneider medium at 32°C for 2 h. All further steps were carried out at room temperature. After three washes in Schneider medium, the cells were fixed for 45 min with 2.5% glutaraldehyde in Schneider medium. They were washed 3 times in phosphate buffer and post-fixed with 1% buffered osmium tetroxyde for 45 min. After 3 washes in phosphate buffer they were maintained in 1% aqueous tannic acid for 15 min and dehydrated in a graded ethanol series. Ethanol was then progressively replaced by Epon resin. The samples were embedded by turning gelatine capsules upside down, directly on the cover slips coated with the cell monolayer. Polymerisation was carried out at 60°C for 24 h. Micrographs were taken at 80 kV on a FEI CM10 transmission electron microscope equipped with an AMT X 60 digital camera (Elexience).

### Statistical Analyses

For purposes of statistical evaluation Student’s t-test for comparing two samples was used. The similarities of deviations were checked with the F-test. The results of the statistical analyses using Student's t-test were considered significant if their corresponding P values were less than 0.05.

## Results

### Plasmids pSci1 to 5-encoded Determinants are Required for Adhesion and Entry of *S. citri* into Ciha-1 Cells

**Figure 1:**
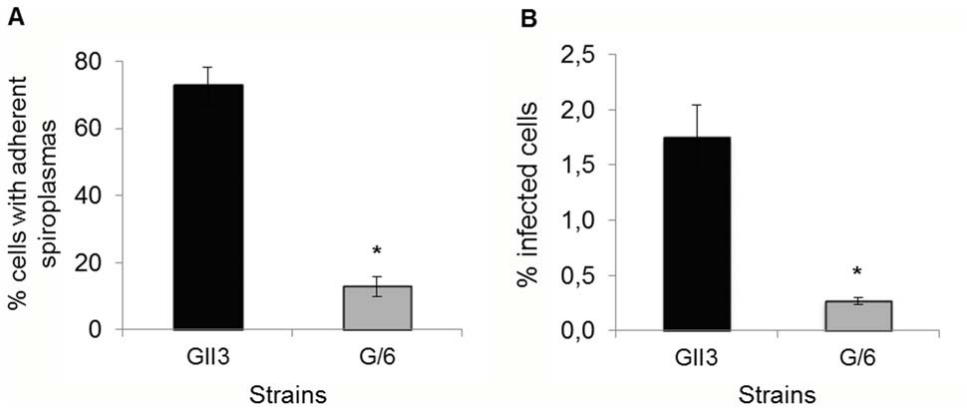
The *S. citri* mutant G/6 is affected in adhesion and invasion of Ciha-1 cells. The insect cells were inoculated with *S. citri* GII3 (MOI 30) and the ScARP-less mutant *S. citri* G/6 (MOI 26). The percentage of cells with adherent spiroplasmas and that of infected cells were determined from CFU counts. Each bar represents mean ± SD of four distinct wells. *, indicates significant differences (Student’s test, P<0.05).

To investigate the role of pSci1 to 5-encoded determinants in invasion of insect cells, the ability of *S. citri* mutant G/6 (lacking pSci1 to 5) to adhere and enter into Ciha-1 cells was compared to that of the wild-type strain GII3. In the experiment of Fig. 1A, adhesion was determined as the percentage of cells with adherent spiroplasmas 4 hours after contact. The results from 4 independent assays clearly showed that the G/6 mutant was significantly less adherent (82% decrease) than the wild-type strain GII3. Similarly, gentamycin protection assays indicated a reduced internalization of *S. citri* G/6 as the percentage of infected cells was significantly lower (84% decrease) as compared to the wild-type strain GII3 (Fig. 1B). These results indicate that pSci1 to 5-encoded proteins probably play a role in adhesion, a critical step towards internalization of spiroplasmas into the leafhopper cells. Amongst these proteins, we postulated ScARPs to be involved.

### ScARPs Localized at the Spiroplasma Cell Surface

**Figure 2:**
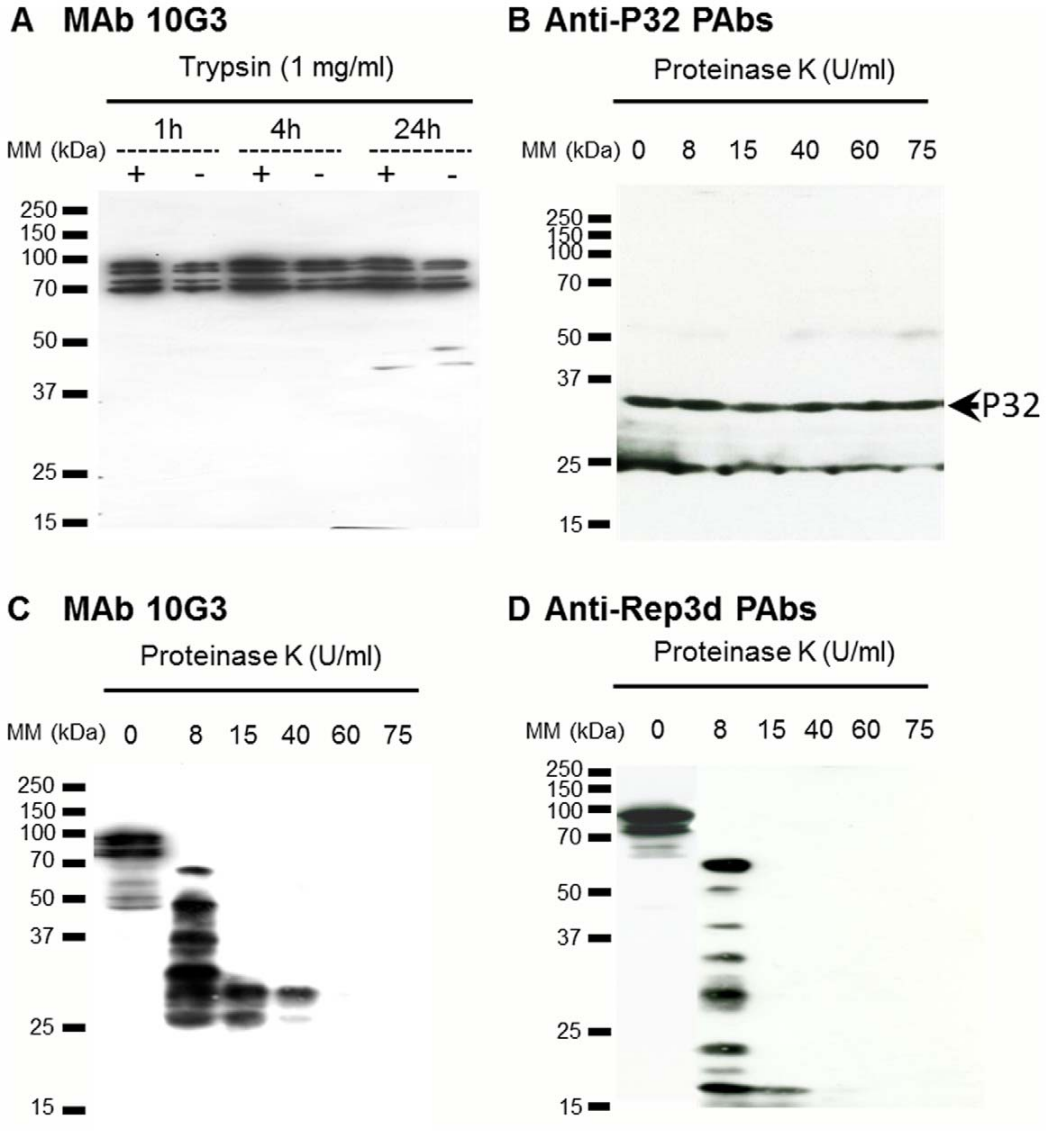
ScARPs are surface exposed. (A) Western immunoblot analysis of ScARPs from *S. citri* GII3. Spiroplasmas were incubated either with the reaction buffer alone (−) or with 1 mg/ml trypsin (+), for 1, 4, and 24 h. Proteins were probed with MAb 10G3. (B–D) Western immunoblot analysis of *S. citri* GII3 treated by proteinase K during 10 min at various concentrations. Proteins were probed with anti-P32 PAbs (B), MAb 10G3 (C), and anti-Rep3d PAbs (D).

Earlier studies showed that ScARPs were amphiphilic, membrane-associated proteins [15], and that ScARPs possessed a signal peptide with a cleavage site and a transmembrane α-helix in the C-terminal region. Thus, the *in silico* analysis of ScARPs sequence suggested that most of the protein was exposed at the spiroplasma surface and hence was potentially involved in the interactions of the spiroplasmas with the host cells. The membrane topology of ScARPs was further investigated through proteolysis and western immunoblot analyses (Fig. 2). Two types of antibodies were used; (i) MAb 10G3, which recognizes an epitope shared by the C-terminal half part of the various ScARPs including ScARP4a [15] and (ii) polyclonal antibodies raised against the recombinant protein Rep3d (see Materials and Methods, Figs. S1 and S2). Anti-Rep3d reacted at least against Rep3d and possibly one more ScARP, according to western blot of Fig. S1C, lane GII3. Proteolysis assays were first carried out using immobilized trypsin at 1 mg/ml. In protein extracts from non-trypsinized spiroplasmas (control) four bands corresponding to uncleaved ScARPs with apparent molecular masses ranging from 75 to 100 kDa were immuno-labelled using MAb 10G3 (Fig. 2A). These proteins were mostly unaffected by trypsinolysis (Fig. 2A). Western immunoblot analysis of proteinase K cleavage products using MAb 10G3 and anti-Rep3d PAbs (Fig. 2C and D) revealed that ScARP3d was mainly surface-exposed. Indeed proteolysis using proteinase K removed the epitopic region recognized by MAb 10G3 (Fig. 2C), as well as the ScARP3d repeat domain (Fig. 2D). Used as a protease-inaccessible control, the cytoplasmic P32 protein [13] was not affected by the proteolytic treatment, as the amount of full-length P32 did not decrease after addition of proteinase K (Fig. 2B). Surface-exposure of the Rep moiety of ScARP3d was further confirmed by darkfield microscopy observations showing that anti-Rep3d polyclonal antibodies induced agglutination of the wild-type spiroplasmas (*S. citri* GII3), while the pre-immune serum did not (Table S1). As expected, no agglutination was detected in the case of the ScARP-less mutant G/6.

### Rep3d Triggers Internalization of Latex Beads Via Actin Polymerization

**Figure 3:**
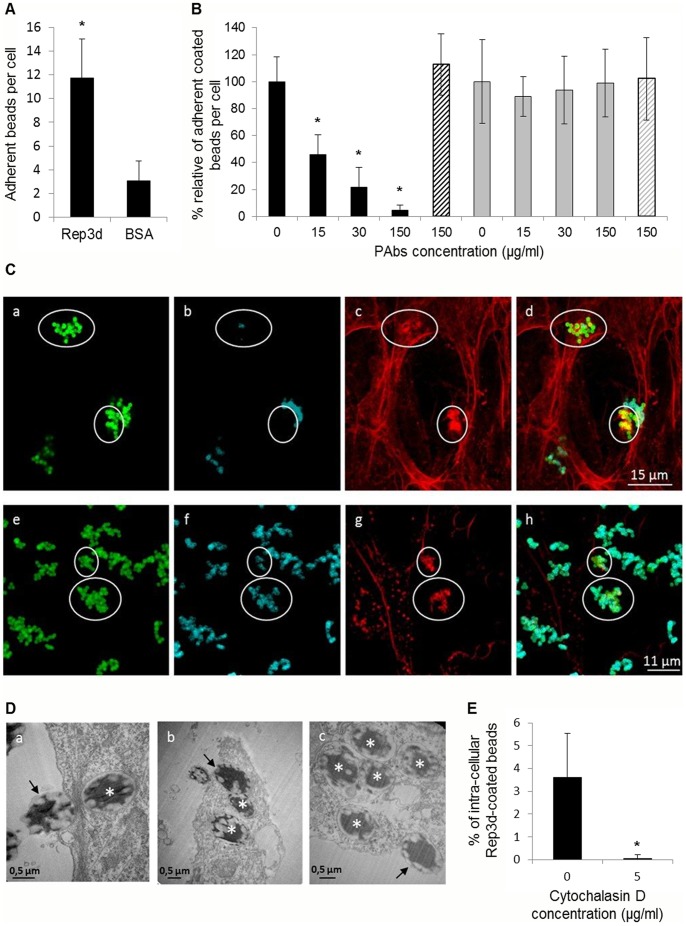
The recombinant protein Rep3d mediates adhesion and entry of latex beads into Ciha-1 cells. (A) The latex beads coated with BSA or Rep3d were incubated for 1 h with Ciha-1 cells on glass slide in 24-well plates. *, indicates significant difference compared to BSA-coated beads (Student’s test, P<0.05). (B) Rep3d-coated beads (black bars) and BSA-coated beads (grey bars) were pre-incubated with anti-Rep3d PAbs or anti-spiralin PAbs (control, hatched bar) at the indicated concentrations before being incubated with Ciha-1 cells as described above. *, indicates significant difference compared to non-treated beads (Student’s test, P<0.05). (C) Double immunofluorescent detection of the Rep3d-coated latex beads attached to and internalized into the Ciha-1 cells before (panels a-d) and after (panels e-h) the permeabilization with 0.2% Triton X-100. Coated beads auto-fluoresced in green (panels a and e), coated beads labeled with anti-Rep3d PAbs were coded in cyan (panels b and f), and actin filaments were coded in red (panels c and g). In the overlays (panels d and h), internalized beads colocalizing with actin appeared yellow. Recruitments of actin filaments at the site of internalized beads are circled (panels c and g, and overlays d and h); circled areas are also indicated in panels a, b, e and f. (D) Transmission electron micrographs of Rep3d-coated beads in Ciha-1 cells. Arrows indicate beads in close contact with the membrane or embedded in the cells; asterisks show internalized beads. (E) Ciha-1 cells were pre-incubated with 5 µg/ml cytochalasin D before addition of Rep3d-coated latex beads. Each bar represents the mean relative percent ± SD of internalized coated beads from two independent replicates. *, significantly different from non-treated cells (Student’s test, P<0.05).

To investigate the ability of the ScARP3d repeat domain to bind leafhopper cells we carried out cytadherence assays with Rep3d or BSA coated latex beads. Fluorescent latex beads were coated with purified Rep3d or BSA (used as the negative control) and incubated with the Ciha-1 cells for one hour before the adherent beads were counted by CLSM observation. As shown in Fig. 3A, the results clearly indicated that the latex beads coated with Rep3d adhered to the Ciha-1 cells surface 4 to 5 fold more efficiently than the control, BSA-coated beads. It should be noted that, in the absence of Ciha-1 cells, the Rep3d- and BSA-coated beads underwent self-aggregation at low and similar rates (1.78±0.22 and 1.26±0.11 beads per aggregate, respectively), limiting a possible bias in adherent beads counts. The adhesion specificity of the Rep3d protein was confirmed by the finding that pre-incubation of Rep3d-coated beads with anti-Rep3d PAbs strongly reduced adhesion to Ciha-1 cells in a dose-dependent manner, whereas anti-spiralin antibodies had no effect (Fig. 3B). In addition, pre-incubation with Rep3d PAbs had no such an effect when applied to latex beads coated with BSA (Fig. 3B).

A permeabilization step was introduced prior to antibody labeling to determine whether the latex beads in close contact with the Ciha-1 cells were internalized or not. Fluorescent latex beads appeared in green in panels a and e of Fig. 3C, due to their intrinsic fluorescence. In the absence of permeabilization, only a subset of the fluorescent beads were labeled by the secondary fluorescent antibodies (Fig. 3C b as compared to a). On the contrary, all of the Rep3d-coated beads were labeled after permeabilization (Fig. 3C f as compared to e). These results clearly indicated that a subset of the coated beads was internalized into the Ciha-1 cells. Internalization of Rep3d-coated beads into Ciha-1 cells was further confirmed by transmission electron microscopy. As shown in Fig. 3D, Rep3d-coated beads were seen in close contact with Ciha-1 membrane as well as embedded (arrows) or internalized (asterisks) inside the cells. Despite apparent clusters of fluorescent beads detected by CLSM microscopy (Fig. 3C), the TEM images clearly showed a single bead (or two in rare cases) per endocytic vesicle (Fig. 3D c), indicating that Rep3d-coated beads were internalized individually. This result is consistent with the finding that self-aggregation of Rep3d-coated beads was not significantly different from that of beads coated with BSA (see above) and thereby exclude the possibility that the number of adherent Rep3d-coated beads (Fig. 3A) might have been overestimated.

Interestingly, actin accumulation was found to occur at the sites where Rep3d-coated beads were internalized (encircled in Fig. 3C c and d), suggesting that rearrangement of actin filaments played a role in the entry of Rep3d-coated beads into the leafhopper cells. To confirm that entry of Rep3d-coated latex beads into Ciha-1 cells occurs through an actin-mediated mechanism, the insect cells were pre-treated with cytochalasin D that inhibits actin polymerization. The percentages of internalized beads in the presence or absence of cytochalasin D were determined from confocal stacks images and compared (Fig. 3E). Despite similar numbers of adherent beads per cell in the presence (3.2±1.7) and absence (3.9±1.9) of cytochalasin D, virtually no bead (0.04% ±0.17) was internalized in the presence of the drug.

### ScARP3d Contributes to Adhesion and Internalization of *S. citri* GII3

**Figure 4:**
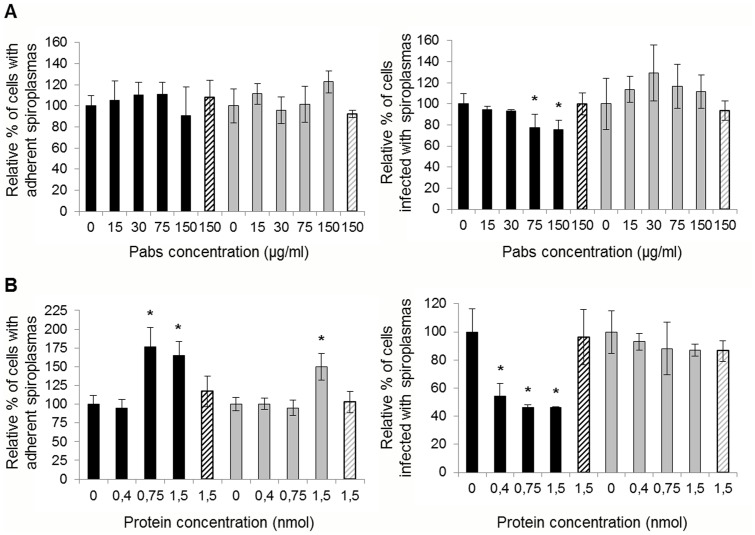
Adhesion and invasion competitive assays with anti-Rep3d PAbs and recombinant Rep3d. (A) *S. citri* GII3 (black bars) and G/6 (grey bars) were pre-incubated with anti-Rep3d PAbs or anti-fibril PAbs (hatched bars) at the indicated concentrations, and then added to Ciha-1 cells at MOI of 65. *, significantly different from *S. citri* pre-incubated in the absence of PAbs (Student’s test, P<0.05). (B) Ciha-1cells were pre-incubated with recombinant protein Rep3d or BSA (hatched bar) at the indicated concentrations before to be inoculated with *S. citri* GII3 (black bars) and *S. citri* G/6 (grey bars) at MOI of 37 and 26, respectively. *, significantly different from Ciha-1 cells pre-incubated with medium alone (Student’s test, P<0.05). Each bar represents the mean relative percent ± SD of four distinct wells.

To determine whether ScARP3d does function as an adhesin, inhibition tests were conducted. In order to prevent fixation of ScARPs to putative cell receptors, spiroplasmas were first treated with various concentrations of anti-Rep3d PAbs prior to be incubated with the leafhopper cells. Previous verifications have shown that, under our experimental conditions, the PAbs did not induce aggregation of spiroplasmas that could have led to false CFU counts (data not shown). The results of adhesion and entry assays are presented in Fig. 4A. Unexpectedly, adhesion of *S. citri* GII3 to Ciha-1 cells was not significantly affected by the treatment with anti-Rep3d antibodies, regardless of the concentration used, and only a slight, dose-dependent decrease of the percentage of infected cells was noticed (Fig. 4A, black bars). As expected the anti-Rep3d antibodies had no significant effect, neither on the adhesion nor on the entry of the ScARP-less mutant G/6 into the Ciha-1 cells (Fig. 4A, grey bars).

To ensure specificity of interaction between ScARP3d Rep domain and putative insect cell receptors, binding competition assays were carried out using recombinant protein Rep3d. Ciha-1 cells were incubated with various concentrations (ranging from 0.4 to 1.5 nmol/well) of Rep3d prior to infection with spiroplasmas. Pre-incubation of insect cells with Rep3d at concentrations higher than 0.75 nmol/well increased adhesion of *S. citri* GII3 to Ciha-1 cells by 76% whereas the same concentrations decreased the spiroplasma entry by 54% (Fig. 4B, black bars). Pretreatment of cells with BSA did neither interfere with adhesion nor with entry of *S. citri* GII3 (hatched bars). It is noteworthy that, with the exception of increased adhesion of G/6 at the higher protein concentration, no such variations were noticed in the case of this ScARP-less mutant G/6 (Fig. 4B, grey bars).

### Actin Polymerization is Required but not Essential for Internalization of *S. citri* GII3

**Figure 5:**
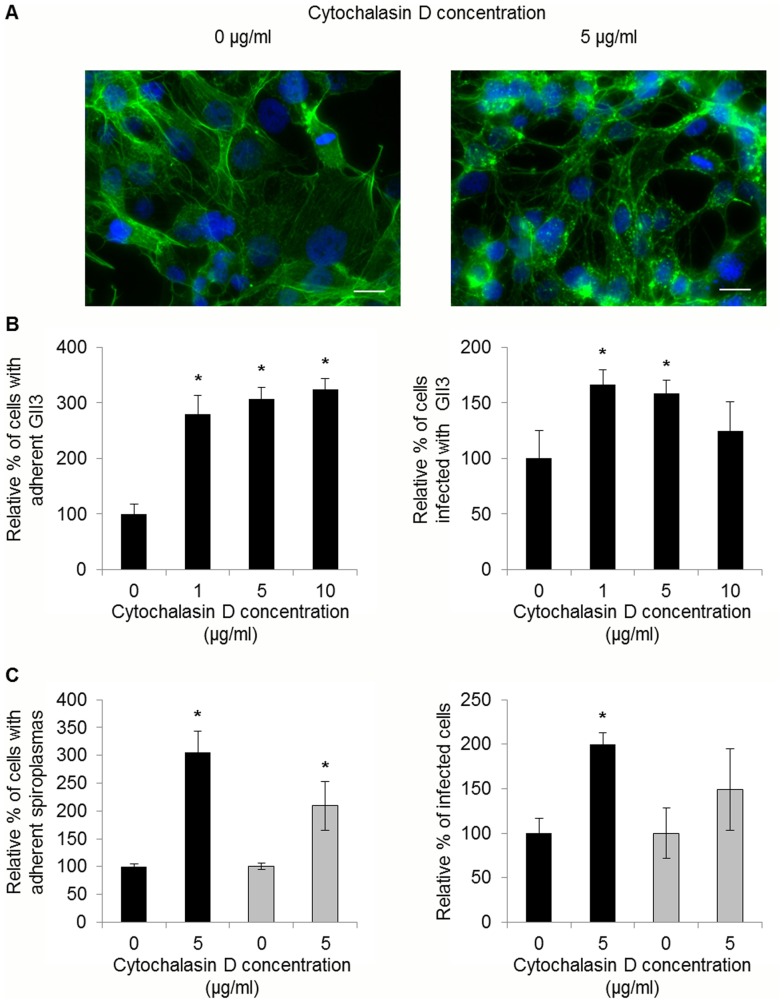
Effect of cytochalasin D on adhesion and entry of *S. citri* into Ciha-1 cells. (A) Ciha-1 cells treated or not with 5 µg/ml cytochalasin D. Actin was stained with Alexa 568-phalloidin (green) and nuclei with DAPI (blue); scale bar, 15 µm. (B) Ciha-1 cells were treated with cytochalasin D for 1 h at the indicated concentrations prior to inoculation with *S. citri* GII3 (MOI 40). *, significantly different from non-treated cells (Student’s test, P<0.05). (C) Ciha-1 cells were inoculated with *S. citri* GII3 (black bars) or the mutant G/6 (grey bars) at MOI of respectively 60 and 65, in the presence or absence of cytochalasin D at the indicated concentrations. *, Significantly different from non-treated cells (Student’s test, P<0.05).

To determine whether spiroplasmas were internalized into Ciha-1 cells through an actin-dependent mechanism, we compared the adhesion and entry of *S. citri* GII3 and the ScARP-less mutant G/6 in the presence or absence of cytochalasin D. As a control we verified that cytochalasin D had no effect on viability (CFU counts), helical morphology, and motility of the spiroplasmas. Fluorescent labeling of Ciha-1 cells with Alexa 568-Phalloidin confirmed that most of actin filaments were disrupted by cytochalasin D treatment (5 µg/ml), increasing separation of adjacent cells as compared to untreated cells (Fig. 5A). Unexpectedly, treatment with cytochalasin D was found to increase adhesion in a dose-dependent manner (Fig. 5B). Entry of spiroplasmas also increased when the cells were treated with 1 µg/ml cytochalasin D. However the percentage of infected cells dropped with increasing concentrations of cytochalasin D (Fig. 5B). Interestingly, whereas 10 µg/ml cytochalasin D treatment boosted adhesion by 3 fold, cell invasion did not significantly increase. As shown in Fig. 5C, increased cytadherence in the presence of cytochalasin D was also detected in the case of *S. citri* G/6, although the increase of cell invasion was not significant in this case.

## Discussion

ScARPs are believed to play a critical role in spiroplasma-host interactions by promoting insect transmission because: (i) ScARPs are conserved in all three plant pathogenic spiroplasmas *S. citri*, *S. kunkelii* and *S. phoeniceum*, as well as in the bee pathogen *S. melliferum* and the rabbit tick spiroplasma sp. 277F [18], [26], [27], [28], (ii) ScARPs are absent in non-insect-transmissible strains of *S. citri* [13], [15] (iii) ScARPs are predicted to localize at the spiroplasma cell surface [14], (iv) ScARPs share homology with *S. citri* BR3 protein P89, the detection of which was associated to adhesion to insect cells [19], and (v) ScARPs share N-terminal repeat domains with characterized adhesins [20]. Repetitive structures of surface proteins are often involved in the interactions of pathogenic bacteria with the eukaryotic host cells [29]. In the porcine pathogen *Mycoplasma hyopneumoniae*, the P97 adhesin (Mhp183) possesses repetitive regions comprising eight tandem copies of a pentapeptide that are required for the mycoplasmas to bind respiratory cilia [30]. Likewise, the TibA adhesin of the enterotoxigenic *E. coli* H10407 contains 17 repeats of 19 amino acids each. These repeats are directly involved in bacterial aggregation and adhesion to the host epithelial cells [31].

Our study focused on the repeat domain of ScARP3d (Rep3d), made of 7 repeats of 38–40 amino acids, which we showed to exhibit adhesin function. Proteolysis results on *S. citri* GII3 using proteinase K confirmed *in silico* predictions, namely that a major part of ScARP3d including the N-terminal repeat domain (Rep3d) was surface-exposed. In addition, despite the high number of Lys residues present throughout its sequence, ScARP3d was unaffected by trypsinolysis, suggesting that this protein could be protected by other surface-exposed proteins and/or steric effects from the protease attack. Spiralin, the major lipoprotein in the membrane of *S. citri* [25] covers most of the membrane surface [32] and was found to be highly resistant to trypsinolysis in earlier *in vitro* studies [33]. Hence, at least *in vitro*, spiralin may form a protective layer at the surface of the spiroplasmal membrane for ScARP3d, and possibly for other surface-exposed antigens, against some possible modes of extracellular degradation. The surface exposure of Rep3d gave ground to the hypothesis that the ScARP3d repeat domain plays an important role in the interactions of the spiroplasma with the leafhopper host cells. Indeed, Rep3d was shown to specifically promote adhesion and entry of latex beads into Ciha-1 cells suggesting that interaction of Rep3d with insect cell receptors was sufficient to induce subsequent endocytosis of the beads. Adhesin-induced internalization of latex beads into the host cells has also been reported for the internalin of *Listeria monocytogenes* [34] and the surface-expressed heat-shock protein Ssa1 of *Candida albicans* [35]. Furthermore inhibition of Rep3d-coated beads entry into Ciha-1 cells by cytochalasin D clearly indicated that actin polymerization was required for endocytosis of the coated beads into these cells as it has been demonstrated for the listeriolysin O of *L. monocytogenes* [36]. Moreover, in contrast to the situation in the wild-type strain GII3, the finding that cytochalasin D treatment had no or little effect on internalization of the ScARP-less mutant G/6 into the Ciha-1 cells, suggests that ScARPs are in some way implicated in the cell invasion process. This result is consistent with those of competitive binding assays showing that adding purified Rep3d to insect cells as well as pre-incubating spiroplasmas with anti-Rep3d PAbs prior to spiroplasmal infection inhibited entry of *S. citri* GII3 into the Ciha-1 cells, despite an increased (with Rep3d) or unchanged (with anti-Rep3d PAbs) adhesion. Similarly, in *E. coli*, competitive binding assays showed that purified intimin promoted adhesion of enterohemorrhagic *E. coli* O157:H7 to HEp-2 cells [37]. To summarize, our data suggest that ScARP3d, like an invasin, could promote entry of the spiroplasmas into the insect cells through attachment to its specific receptor but the mechanism remains unknown. Based on literature, several mechanisms, including modification of ScARPs and/or their receptors through phosphorylation, cleavage and oligomerization, can be hypothesized to enable host cell invasion. In uropathogenic *E. coli*, the binding of adhesin FimH to its receptor induces conformational changes of the entire receptor complex that trigger signal transduction, enabling entry into the host cells [38], [39]. In the malaria parasite *Plasmodium falciparum*, cleavage of a variety of adhesins by membrane proteases was shown to be essential for mosquito midgut and salivary gland invasion [40]. Other studies in *Yersinia pseudotuberculosis* demonstrated that invasin homotypic interaction was required for efficient uptake by the host cell [41].

Surprisingly, adhesion of spiroplasmas strongly increased in the presence of cytochalasin D, an inhibitor of actin polymerisation. In *Salmonella typhimurium* and *Staphylococcus aureus*, it has been shown that cytochalasin D treatment of polarized human enterocytes or mouse renal cells resulted in the exposure of hitherto latent receptors [42], [43]. Therefore, though it is unclear why the cytochalasin D treatment increased adhesion of both *S. citri* GII3 and G/6 to Ciha-1 cells, one may speculate that, by opening tight junctions, cytochalasin D might reorganize the membrane proteins at the cell surface, uncovering new membrane receptors for spiroplasma adhesins other than ScARPs. This could explain why adhesion of the Rep3d-coated beads did not increase when treating cells with cytochalasin D. The finding that a Rep3d concentration of 1.5 nmol/well increased adhesion of both *S. citri* GII3 and G/6 also suggested that the physical association of the Rep domain to Ciha-1 cell proteins could further enhance adherence of spiroplasmas, not only via ScARPs but also via one or several additional adhesins. Thus, it is likely that adhesion of *S. citri* to Ciha-1 cells involves several spiroplasmal proteins, similarly to *M. hyopneumoniae* which binds respiratory cilia and heparin via a large set of adhesins [44], [45], [46], [47]. This hypothesis is reinforced by the fact that the ScARPs-less mutant G/6 is still insect-transmissible, though at low efficiency [17]. Moreover, successful transmission requires the spiroplasmas to invade various cell-types including the midgut epithelium and the salivary gland cells, which receptors probably recognize distinct spiroplasmal proteins. One of these could be spiralin, the major membrane lipoprotein, which is known to act *in vitro* as a lectin binding glycoproteins of the leafhopper vector [48]. Moreover it has been reported that *S. citri* undergoes morphological changes from helical to rounded shape when attaching to insect cells *in vivo* [8], [11] as well as *ex vivo* [12]. Whether and how the fibril and MreB cytoskeleton proteins of the spiroplasmas are reorganized during these early events of insect cell invasion is still to be investigated. Such major morphological changes certainly involve a broad set of protein-protein interactions and/or modifications (*e. g*., phosphorylation) that are induced by adhesion-receptor interactions. In previous studies searches for such interactions using *in vitro* protein overlay assays revealed that entry of *S. citri* into Ciha-1 cells involved interaction between the spiroplasmal phosphoglycerate kinase and actin of the leafhopper cells [23].

In *S. citri*, de novo actin polymerisation is required for efficient internalization of spiroplasmas into the Ciha-1 cells as increasing concentrations of cytochalasin D resulted in a decrease of spiroplasma entry. Consistent with these results, spiroplasmas have been shown to co-localize with actin filaments in salivary gland cells of *S. citri* infected leafhoppers [23]. However, in the present study entry of spiroplasmas into Ciha-1 cells was not fully abolished by cytochalasin D, suggesting that other cytoskeleton components were involved in endosome formation. Clathrin-dependent endocytosis has been implicated in the internalization of various pathogens [49]. Whether such a mechanism could promote entry of *S. citri* into Ciha-1 cells has to be examined.

Interestingly, analysing the Rep3d sequence with the CD-Search service [50] identified a ligand-binding sensor conserved domain involved in bacterial signal transduction (COG3292). Together with the detection of cleavage products [15], these results suggest that besides being adhesins the *S. citri* ScARPs might play diverse functions when interacting with the insect host cells. Eventually, despite they share strong homology, the eight *S. citri* ScARPs differ by the number of repeats and the charged amino acids at the C-terminal end so that the possibility that they play distinct roles cannot be excluded.

## Supporting Information

Figure S1 **Purification of Rep3d recombinant protein and detection of ScARPs from** ***S. citri*** **whole cell extracts.** (A) Coomassie-stained 12.5% SDS-PAGE gel of purified Rep3d from *E. coli*. P1, pellet; S1, supernatant; LW1, washing; E1-1 to E3-1, eluted fractions following the first Nickel affinity column; PD10, eluate from PD-10 column; E1-2 to E3-2, eluted fractions following the 2nd Nickel affinity column; 3 µg and 4 µg, µg of BSA control; Rep3d, Rep3d recombinant protein. (B) Coomassie-stained 12.5% SDS-PAGE gel of purified Rep3d before (left arrow) and after (right arrow) cleavage with thrombin. (C) Western immunoblot analysis of whole cell lysates of *S. citri* GII3 (GII3) and *S. citri* ScARP-less G/6 (G/6), purified His-tagged Rep3d (His_6_-Rep3d), and purified Rep3d without poly-His tag (Rep3d). Proteins were probed using anti-Rep3d PAbs.
(TIF)

Figure S2 **Amino acid sequence of ScARP3d.** Amino acids in italics indicate the predicted signal peptide. The Rep3d fragment expressed in *E. coli* is highlighted in grey. In this fragment, the repeats are indicated by arrows and the predicted transmembrane segment is boxed.
(TIF)

Table S1 **Induction of spiroplasmas agglutination by anti-Rep3d PAbs.**
(DOC)
