# Supplementary figures and images for: The Repetitive Domain of ScARP3d Triggers Entry of Spiroplasma citri into Cultured Cells of the Vector Circulifer haematoceps
Source: PLoS One. 2012 Oct 31;7(10):e48606. doi: 10.1371/journal.pone.0048606 (PMC3485318; doi:10.1371/journal.pone.0048606)

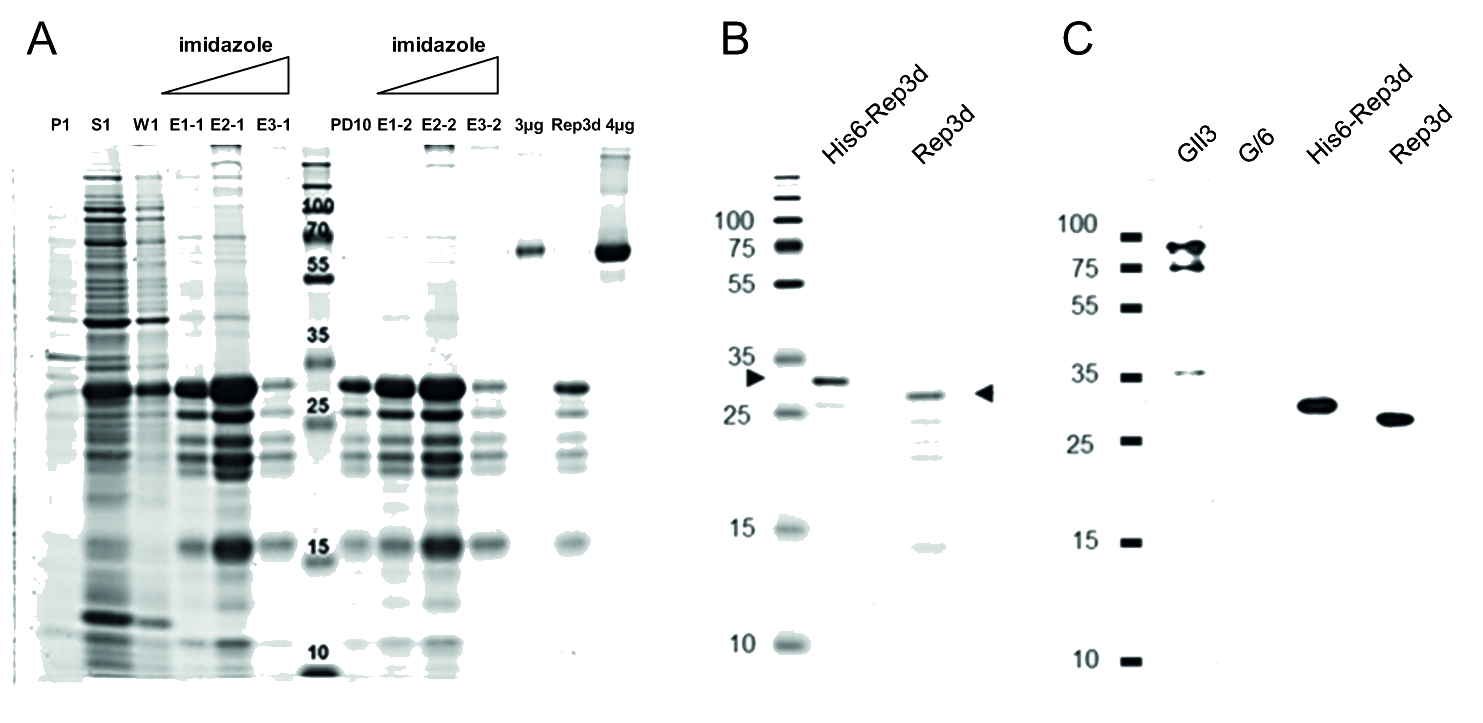

Supplement: Figure S1 — Purification of Rep3d recombinant protein and detection of ScARPs from S. citri whole cell extracts. (A) Coomassie-stained 12.5% SDS-PAGE gel of purified Rep3d from E. coli. P1, pellet; S1, supernatant; LW1, washing; E1-1 to E3-1, eluted fractions following the first Nickel affinity column; PD10, eluate from PD-10 column; E1-2 to E3-2, eluted fractions following the 2nd Nickel affinity column; 3 µg and 4 µg, µg of BSA control; Rep3d, Rep3d recombinant protein. (B) Coomassie-stained 12.5% SDS-PAGE gel of purified Rep3d before (left arrow) and after (right arrow) cleavage with thrombin. (C) Western immunoblot analysis of whole cell lysates of S. citri GII3 (GII3) and S. citri ScARP-less G/6 (G/6), purified His-tagged Rep3d (His6-Rep3d), and purified Rep3d without poly-His tag (Rep3d). Proteins were probed using anti-Rep3d PAbs. (TIF) [file pone.0048606.s001.tif]

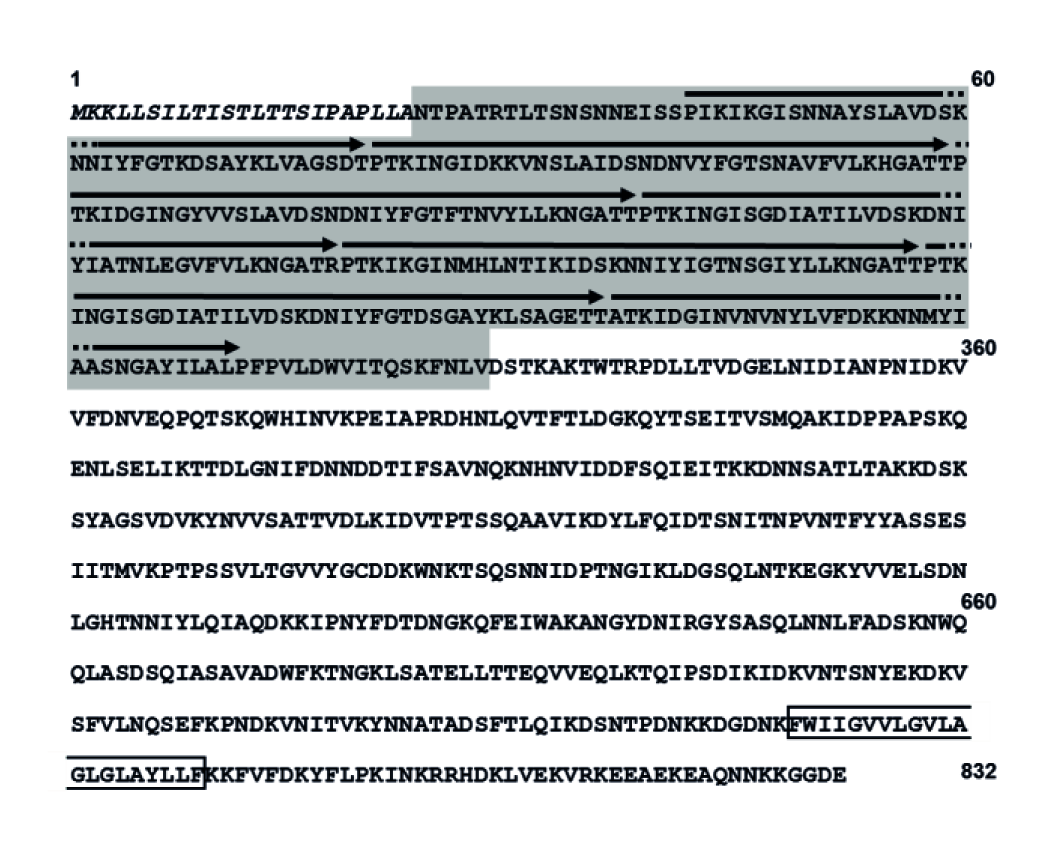

Supplement: Figure S2 — Amino acid sequence of ScARP3d. Amino acids in italics indicate the predicted signal peptide. The Rep3d fragment expressed in E. coli is highlighted in grey. In this fragment, the repeats are indicated by arrows and the predicted transmembrane segment is boxed. (TIF) [file pone.0048606.s002.tif]
