# Supplementary material for: The Repetitive Domain of ScARP3d Triggers Entry of Spiroplasma citri into Cultured Cells of the Vector Circulifer haematoceps
Source: PLoS One. 2012 Oct 31;7(10):e48606. doi: 10.1371/journal.pone.0048606 (PMC3485318; doi:10.1371/journal.pone.0048606)
Supplement: Table S1 — Induction of spiroplasmas agglutination by anti-Rep3d PAbs. (DOC) [file pone.0048606.s003.doc]

Supplemental Table S1. Induction of spiroplasmas agglutination by anti-Rep3d PAbs.

| Antibodies | *S. citri* strain | AC_50_ (mg/ml) |
| --- | --- | --- |
| Anti-spiralin PAbs | GII3 | 0.05 |
| Anti-spiralin PAbs | G/6 | 0.05 |
| Anti-Rep3d PAbs | GII3 | 0.2 |
| Anti-Rep3d PAbs | G/6 | - |
| Pre-immune serum | GII3 | - |
| Pre-immune serum | G/6 | - |

*AC_50_*, immunoglobulin concentration agglutinating 50% of spiroplasmas. Data are representative of three independent experiments.
